# Valorization of Red Beetroot (*Beta vulgaris* L.) Pomace Combined with Golden Linseed (*Lini semen*) for the Development of Vegetable Crispbreads as Gluten-Free Snacks Rich in Bioactive Compounds

**DOI:** 10.3390/molecules29092105

**Published:** 2024-05-02

**Authors:** Julia Niemira, Sabina Galus

**Affiliations:** Department of Food Engineering and Process Management, Institute of Food Sciences, Warsaw University of Life Sciences, Nowoursynowska Str. 159c, 02-776 Warsaw, Poland; s202091@sggw.edu.pl

**Keywords:** beetroot, linseed, crispbread, vegetable snack, bioactive compounds

## Abstract

This work aimed to develop gluten-free snacks such as crispbread based on beetroot pomace (*Beta vulgaris* L.) and golden linseed (*Lini semen*). Beetroot is attracting more and more consumer attention because of its nutritional and health properties. The use of beet pomace contributes to waste management. Linseed, known as a superfood with many health-promoting properties, was used to produce crispbreads as an alternative to cereals, which are allergens. Beetroot pomace and whole or ground linseed were used in different proportions to produce crispbread snacks. Chemical and physical analyses were performed including water activity, dry matter, betalains, and polyphenols content, as well as Fourier transform infrared spectroscopy (FTIR). A sensory evaluation and microstructure observations were also performed. The obtained snacks were characterized by low water activity (0.290–0.395) and a high dry matter content (93.43–97.53%), which ensures their microbiological stability and enables longer storage. Beetroot pomace provided betalains—red (14.59–51.44 mg betanin/100 g d.m.) and yellow dyes (50.02–171.12 mg betanin/100 g d.m.)—while using linseed enriched the product with polyphenols (730–948 mg chlorogenic acid/100 g d.m.). FTIR analysis showed the presence of functional groups such as the following: -OH, -C-O, -COOH, and -NH. The most desired overall consumer acceptability was achieved for snacks containing 50% beetroot pomace and 50% linseed seeds. The obtained results confirmed that beetroot pomace combined with linseed can be used in the production of vegetable crispbread snacks.

## 1. Introduction

Vegetables are a significant part of the human diet and provide many nutrients—including fiber, vitamins, and minerals [1]. Vegetables can be divided into root vegetables, legumes, and fruits. Root vegetables such as carrots, beetroots, and parsley are good sources of fiber, are low in calories, and contain many bioactive compounds [2,3]. Because of the prevailing trends related to healthy food and its bioactive potential, beetroot is attracting more and more attention from researchers and consumers [4,5]. Beetroot (*Beta vulgaris*) belongs to the *Chenopodiaceae* family, which includes nearly 1400 plant species and has several varieties that can range in color from yellow to red. Nevertheless, the most consumed beetroot is red. This kind of beetroot has been used in the human diet for centuries for its nutritional and health properties [6]. The literature increasingly reports the presence of many bioactive compounds such as phenolic compounds and betalains, which could classify beetroot as a functional food [5,7]. In addition, this vegetable is an excellent source of minerals and vitamins that ensure the proper functioning of the human body [3]. Beetroot tubers are a good source of zinc, manganese, and phosphorus, while large amounts of iron, potassium, and sodium accumulate in the leaves. Beetroot roots are rich in vitamins C, A, E, and K. The presence of B vitamins improves memory and increases blood flow to the brain. Ingredients in beetroots help treat anemia and reduce blood pressure [8]. Moreover, beetroot is a good source of betalains, which are hydrophilic pigments that can be divided into two groups as follows: betacyanins, which consist of betalamic acid and cyclo-3,4-dihydroxyphenylalanine, characterized by a reddish-purple color, and betaxanthins, which consist of an amino acid combined with betalamic acid, characterized by a yellow to orange color [9]. The concentration of betalains in red beetroots is approx. 120 mg per 100 g of fresh weight and depends on the species, growing conditions, storage method, and the part of the vegetable used (more in the peel) [10]. Betalains are important for human health because of their antioxidant, anti-inflammatory, and antimicrobial properties [11]. In addition to betalains, the antioxidant properties of beetroots are also influenced by the presence of phenolic compounds, which include phenols and flavonoids [12].

Beetroots are usually consumed after heat treatment in the form of soups and less often in raw form (components of vegetable mixtures). They require peeling, and their consumption is not high, especially among young people. However, beetroots are used for fresh-pressed juices, and this process results in waste, which, when produced in large amounts, is a major problem in the food industry. An important aspect is to manage leftovers to avoid environmental pollution. Waste from vegetable and fruit processing can be used as a source of bioactive compounds [13]. In the case of beet, these compounds are mostly betalains or polyphenols [14]. Beet pulp is also used in powder form as cheap, calorie-free food fillers, e.g., instead of flour, because of the high content of fiber and high capacity to retain oil or water [15]. After the juice is squeezed out, about 15 to 30% of the pomace remains and, despite its high potential, the pomace is discarded or rarely used as animal feed [14]. A good alternative is the use of pomace in the food industry as an ingredient in different products such as cookies, candies, and crispbreads. With the presence of beet pomace, an increase in fiber content and antioxidant activity has been noted [16,17].

Linseed (*Linum usitatissimum*) is a blue-flowered plant that produces small and flat seeds. They can range in color from golden yellow to reddish brown depending on the variety. The golden seeds of linseed have a nutty and buttery flavor, which is why they are commonly consumed in the following three ways: whole, ground, and linseed oil [18]. Linseed is referred to as a superfood because of its properties that positively affect human health such as regulating intestinal flora, alleviating nervous disorders, and supporting circulation [19]. The addition of linseed to foods improves their nutritional value because of the presence of bio-components such as proteins, fibers (soluble and insoluble), and fats [20]. Linseed contains from 3 to 10% mucilage in the whole seed weight, which is characterized by health benefits, such as lowering glucose levels [21]. The mucilage derived from linseed is hydrophilic, and the mucilage’s functionality is similar to gum Arabic. Both exhibit good rheological properties and water-binding capacity. The use of mucilage in the food industry depends on its functional properties; in the case of hay, mucilage can be used as a gelling or thickening agent [22]. Linseed is also considered a very good source of protein because of the presence of sulfur amino acids such as cysteine and methionine; branched-chain amino acids such as leucine, isoleucine, alanine; and essential amino acids such as tyrosine, threonine, and lysine [23]. Linseed proteins exhibit many functional characteristics such as their ability to form emulsions and foam and their ability to absorb water [24]. Moreover, the presence of phenolic compounds (approx. 1030 mg/100 g of phenolic acids), minerals (phosphorus, calcium, and magnesium), and vitamins have been shown to have health properties such as antimicrobial and anti-inflammatory effects [25].

One of the most popular types of snacks is dried and extruded products, which are very crunchy and can take different shapes. Extrusion involves plasticizing raw materials under high pressure, high temperature, and shear forces, and then the material is extruded through a forming nozzle [26]. Among these types of snacks are crispbreads and crackers, which have recently gained a lot of interest because of their health benefits and long shelf life. Crispbreads are used as a substitute for bread for sandwiches but also make an attractive snack on their own. Nowadays, crispbreads can be found on the market made with rye, rice, and wheat flour with additives such as tomatoes, basil, sesame, and beetroot. Most additives are used in dry or freeze-dried form. Cereal and gluten-free products such as cakes, cookies, and extruded snacks are rich in carbohydrates and protein but are low in fiber, minerals, and vitamins. Adding beetroot to daily snacks provides nutrients and meets nutritional needs. Lucky et al. [27] studied the effect of added beetroot powder on the nutritional value of cake. Their study showed that the addition of beetroot powder significantly increased the amount of protein, ash, and fiber in the final product. Lisiecka et al. [28] studied the effect of fresh beetroot pulp addition on the properties of gluten-free rice snacks. The addition of pulp increased the total phenolic content, which may be a good source of antioxidants that are important for human health. Abdo et al. [29] produced cookies with the addition of powder obtained from beetroot pulp. Their analysis showed that the addition of beetroot powder increased fat, ash, and fiber content. The cookies were also characterized by increased calcium, magnesium, or potassium content. Because of the easy availability of sweet cereal snacks in stores, the addition of beetroot would enrich the diets of today’s consumers.

In general, snacks are mainly consumed between meals and often contain food additives that do not provide a clean label. Adding linseed to snacks is a good way to provide nutrients and thus enrich the human diet. Yuksel et al. [30] made wheat chips with linseed flour addition at 10, 15, and 20%, which increased the dry matter, protein content, and unsaturated fatty acids of the product. Similar observations were noted by Subedi and Upadhyaya [31] for cereal bars incorporated with linseed flour. In addition, the bars based on oats were significantly better than the sample without linseed flour in terms of texture and overall acceptability. Because of the high consumption of different snacks, the use of vegetables and linseed as raw materials will reach many consumers. Moreover, the application of agricultural by-products in the design of new food products is in line with the circular approach and meets the expectation of plant-based sustainable food. Therefore, this work aimed to develop and characterize the selected physical properties of gluten-free vegetable snacks based on beetroot pomace and linseed.

## 2. Results and Discussion

### 2.1. Vegetable Crispbread Characterization and Microstructure

**Figure 1:**
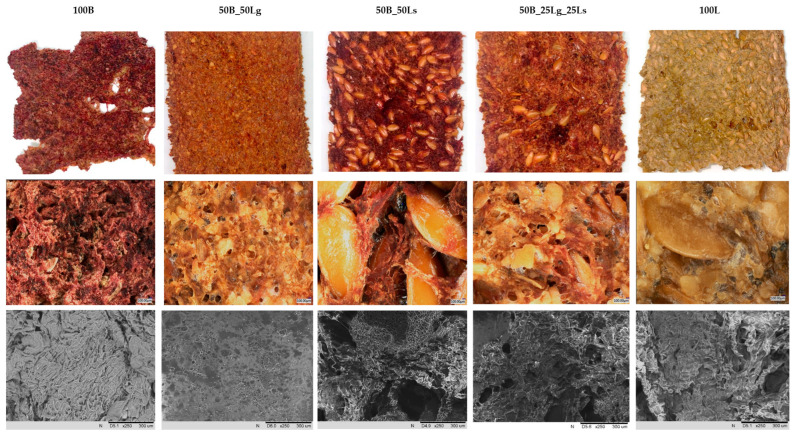
Digital and microstructure photographs taken under a Keyence microscope and a scanning electron microscope of the vegetable crispbreads based on beetroot (B) pomace and linseed (L).

Several studies have been carried out on the development of new forms of products or packaging films and coatings based on fruits and vegetables. This kind of investigation relies mostly on combining different kinds of film-forming hydrocolloids (i.e., polysaccharides or proteins) with fruit and vegetable purées [32,33]. On the other hand, some fruits and vegetables are a source of nutrients and antioxidants that may be ingested in the form of edible films made from them. However, there is also a possibility to prepare composite materials based on the mixture of multiple vegetables and use them as a wrap or edible paper for food application. Nevertheless, most research is still at the laboratory stage, especially regarding by-products from processing. It can be noted that some fruits contain enough pectins to make it possible to produce continuous materials without the addition of gelling agents, such as apple, black currant, or plum [34]. However, vegetables are characterized by different compositions, and the beetroot pomace produced during juice processing that was used in this study did not contain a sufficient amount of gelling compounds to create thin layers of vegetable snacks (Figure 1). Therefore, striving for a clean label and desiring to design a natural new product, linseed as a mucilage-producing component was used in the form of whole or ground seeds to form a natural vegetable snack. The prepared crispbreads were in different forms including pure beetroot pomace (100B) and pure linseed samples (100L) combined with the mixture of both ingredients in the proportion of 50:50 containing linseed as ground (50B_50Lg) or whole seeds (50B_50Ls), as well as the combination of both forms (50B_25Lg_25Ls). A significant difference was observed among the samples resulting from their composition in the context of color, texture, and surface of the vegetable crispbreads. There was a similar thickness among the samples, between 3.5 ± 0.5 mm, for those containing beetroot pomace and linseed regardless of the component forms, and 5 ± 1 mm, for pure linseed snacks. Pure beetroot snacks had a thickness of 4 ± 1 mm. All samples, except pure beetroot pomace snacks, were characterized by relatively uniform surfaces because of the presence of linseed mucilage, which played a role as a natural gelling and structure-forming agent. Linseed contains approx. 6% mucilage, a non-starch polysaccharide, which is predominantly (75%) composed of arabinoxylans. During the initial phase of baking, water-extractable arabinoxylans stabilize gas cells and improve material characteristics such as firmness, structure, and texture. Therefore, it is possible to improve the structure of the product by incorporating a small amount of water-soluble arabinoxylans from linseed mucilage [35].

The surface of pure beetroot snacks (100B) showed a lot of pores, which can influence, among other things, the increased hygroscopicity of the product. In the case of pure linseed snacks (100L), mucilage is visible on the surface in addition to the seeds and ground linseed, which is a binder for the snacks tested and therefore reduces their porosity. In the case of the sample containing ground and whole linseed (50B_25Lg_25Ls), both forms are visible on the surface. The beetroot pomace is bonded with linseed mucilage, and the porosity is reduced, but there is less mucilage produced compared with the snacks containing full ground linseed (50B_50Lg). This variant is more consistent and cohesive in comparison with the others because of the good miscibility and compatibility of both components in similar particle sizes. For all snacks, a significant visible change in color was observed, from dark purple for pure beetroot pomace snacks to yellow for pure linseed snacks as a result of the characteristics of each component. Observations of the crispbread microstructure using optical and scanning electron microscopes showed the arrangement of the beetroot pomace fibers and the porosity of the materials. In addition, in the case of pure linseed snacks (100L), dark patches that correspond to fat are visible. The surface of all analyzed snacks is irregular and rough, which is typical for dried vegetable materials because of the porous character of the materials and the formation of the structure during the hot air drying process [36].

### 2.2. Color

Food color plays a key role in consumers’ perceptions of the quality and attractiveness of food products. It is one of the factors influencing purchasing decisions. Food color can be affected by different parameters such as water content, heat treatment, or quality changes associated with, for example, oxidation during storage [37]. The results of the color parameters for vegetable crispbreads are presented in Table 1. The L* parameter determines the lightness of the test sample and was in the range of 32.82–53.30. The higher the value of the L* parameter, the lighter the color. Statistically significant differences were observed between samples containing beetroot pomace, linseed, and a combination of both components (*p* < 0.05). The lowest value for parameter L* (32.82 ± 1.90) was obtained for the pure beetroot crispbreads (100B), while the highest (53.30 ± 1.75) was obtained for the pure linseed crispbreads (100L). This is attributed to the original color of the ingredients, dark purple and yellow. The addition of whole seeds or ground linseed increased the lightness of the crispbreads. The sample containing 50% ground linseed (50B_50Lg) had one of the higher values of this parameter (39.41 ± 1.37). This may be due to the form of linseed used. The milling of the seed enabled it to be thoroughly combined with the marc throughout the mass. The sample with the addition of 50% seeds (50B_50Ls) had a lower value (37.93 ± 2.26) of lightness compared with the sample with ground linseed, which could be due to the uneven distribution of the seeds. For the sample with the mixture of both forms of linseed (50B_25Lg_25Ls), the lightness was relatively higher than the other samples (45.03 ± 2.76), which could be due to the formation of clusters of ground seeds. In addition, during thermal processing, the snacks were browned, resulting in lower lightness. The brown color may have been due to the Maillard reaction occurring on the surface of the snack, where reducing sugars and proteins reacted [38].

Parameter a* represents green-red shades, and positive values indicate colors toward red. For the analyzed vegetable crispbreads, values of parameter a* were positive and in the range 7.97–21.16 (Table 1). Statistically significant differences were observed between the samples containing beetroot pomace, linseed, and a combination of both components (*p* < 0.05) with the tendency to decrease in value being associated with the addition of linseed. The highest value of the a* parameter (21.16 ± 1.32) was achieved by the pure beetroot crispbreads (100B) and the lowest value (7.97 ± 0.55) by the pure linseed crispbreads (100L). All snacks containing pomace and flaxseed showed relatively high a* parameter values in the range of 16.66–18.01, which may be due to the high extraction of betalains from beetroot tissue as a result of the high heat treatment [39]. The addition of ground linseed (50B_50Lg), and thus carotenoids, provided the lowest value (16.66 ± 0.62) for this parameter because of the uniform distribution of this raw material throughout the pulp. In the case of the addition of whole linseed (50B_50Ls), the red color was better preserved (18.01 ± 1.62) because of the formation of seed clusters that were unevenly combined with the pomace and, consequently, the color was not altered throughout the material. Beetroot owes its intense purple color to the presence of pigments—betalains [9]. In the case of linseed, its yellow-gold color is due to the presence of carotenoids [40].

Parameter b* represents blue-yellow shades, and positive b* values, which can be observed for the analyzed vegetable crispbreads (Table 1), indicate colors toward yellow. The values were obtained in the range of 11.54–24.72, and statistically significant differences were observed among the samples containing beetroot pomace, linseed, and a combination of both (*p* < 0.05). The highest value of this parameter (24.72 ± 1.09) was achieved by the pure linseed crispbreads (100L) and the lowest (11.54 ± 1.42) by the pure beetroot crispbreads (100B), which corresponds with the characteristic of each component. The addition of linseed to the beetroot crispbreads caused a color change toward yellow, which is due to the presence of carotenoids [40]. This increased parameter b* (23.15 ± 0.71) for the sample containing ground linseed (50B_50Lg), which, as in the previously described color parameters, depended on the uniform distribution of the ingredients in the snacks. Samples 50B_50Ls and 50B_25Lg_25Ls had lower values of parameter b* at 15.90 ± 1.74 and 16.30 ± 2.26, respectively, which may be due to the non-uniform distribution of the linseed. The yellow color may also be affected by the cleavage of betanin into bright yellow balsamic acid and colorless cyclodopa-5-O-β-glucoside during thermal processing [41].

### 2.3. Dry Matter

The higher the dry matter content and, consequently, the lower the water content, the greater the stability of the product. Therefore, knowing the dry matter content of a product is crucial to determining the stability and shelf life of a food product. A low water content limits the occurrence of quality changes such as microbial growth [42]. The dry matter content of the crispbread snacks ranged from 93.42 to 97.53% (Table 2). Evaporation of water yielded a beetroot pomace snack (100B) with a high dry matter content of 93.43 ± 0.48%, while the linseed snack (100L) had significantly higher dry matter content (97.1 ± 1.41%). Statistically significant differences were observed between the sample containing beetroot pomace and all samples with linseed (*p* < 0.05).

The addition of linseed to beetroot pulp resulted in an enrichment in the snacks in terms of dry matter. The highest dry matter value (97.53 ± 0.20%) was recorded for sample 50B_50Lg, which could be due to the presence of ground linseed, which provided a large surface area for water evaporation, similar to sample 50B_25Lg_25Ls (97.4 ± 0.06%). The lowest dry matter content (97.04 ± 0.08%) was found in sample 50B_50Ls because of the presence of whole seeds, which may have hindered water evaporation. However, all the values are relatively high, indicating the very low water content of the obtained vegetable snacks with the tendency of lower values for beetroot pomace. This is attributed to the high water content of fresh beetroot pulp, approx. 85–90% [43], which does not ensure the stability of the material; thus, it should be preserved. Dehghan-Manshadi et al. [44] noted the dry matter of linseed before drying to be 86.2% and after drying to be 95% (105 °C). Similar results for dry matter (91.8%) were obtained by Sahni and Shere [45] for beetroot pomace. A low moisture content in pomace is crucial to maintain good stability; thus, the analyzed vegetable crispbreads based on beetroot pomace and linseed may be recognized as a stable dry product because of the very low water content (below 3%). These observations are in line with others related to crispbreads as products with low water contents (3–10%), long shelf lives, and crispness [46].

### 2.4. Water Activity

One of the parameters that well characterizes food stability is water activity. This parameter allows for the presence of water in the product to be linked to the properties, quality, and stability of the food. It is assumed that below a water activity of 0.6, no microorganisms can grow and that below 0.8, most enzymatic reactions are inhibited. The minimum rate of fat oxidation occurs at water activities of 0.2–0.35, and enzymatic browning increases in intensity with increasing water activity. The greatest stability of food products is achieved when the water activity is at the level of the water content of the monolayer, which is between 0.07 and 0.35 [47]. The water activities of both linseed and ground linseed were below 0.6, at 0.577 and 0.596, respectively. This translates into a lower water content and higher dry matter content compared with the raw beetroot pomace, which was characterized by a high water activity of 0.995. During the vegetable snack preparation, the water activity of the materials was measured before and after the thermal treatment (baking at 160 °C), and the results are presented in Table 3.

The increased temperature contributed to the evaporation of water; thus, a reduction in water activity was observed. Before baking, each sample had a very high and similar water activity in the range of 0.994–0.997, which did not ensure their microbiological stability or shelf life. After baking, the snacks reached a water activity value in the range of 0.290–0.395, which prevented microbial growth and enzymatic reactions and significantly increased their stability. The water activity decreased by a factor of three, and it is known that as water activity decreases, shelf life increases. Statistically significant differences were observed in the samples containing beetroot pomace, linseed, and a combination of both ingredients (*p* < 0.05). The pure beetroot crispbread sample (100B) achieved the highest water activity value (0.395 ± 0.001), which is due to beetroot being a high-moisture vegetable and the structure of pomace, which may cause limited water evaporation during drying. The pure linseed crispbread sample (100L) achieved a value of 0.317 ± 0.005. The sample containing 50% ground linseed (50B_50Lg) had the lowest value (0.290 ± 0.009) of water activity, while the sample containing 50% linseed (50B_50Ls) had a water activity value of 0.342 ± 0.003. This tendency is by the value of 310 ± 0.002 obtained for the sample containing linseed as a mixture of whole and ground seeds (50B_ 25Lg_25Ls). The lower water activity of ground linseed may be due to the increased surface area obtained during milling, which allows for greater water evaporation. Conversely, whole seeds have a smaller evaporation area as the water is enclosed within the seed coat, resulting in higher water activity. Similar patterns were observed by Gondek et al. [48], who studied the physical properties of gluten-free crispbread. The subjects of their study were extruded bread made of corn porridge without additives and cornbread with the addition of rice flour, buckwheat flour, and amaranth seed flour. The water activity of the crispbreads ranged from 0.25 to 0.35, corresponding to water content in the range of 7.12–8.33/100 g d.m. Marzec and Lewicki [49] reported that the water content of those values is close to the water content of the monolayer, which is the optimum value for preserving the storage stability of the product. The analyzed vegetable snacks based on beetroot pomace and linseed showed water activity in this range, which also ensures their storage stability. Konrade et al. [50] developed crispbread snacks based on by-products from apple juice production and rice flour. The water activity of the extruded snacks ranged from 0.45 to 0.49, which is higher than the values obtained in this study (0.290–0.395) and indicates that components and the baking process affect the water activity values of the products.

### 2.5. Water Vapor Adsorption Kinetics

The sorption of water vapor by the product is of great practical importance related to subsequent storage. If a product is stored unpackaged in an environment having a relative humidity higher than the equilibrium relative humidity of the product, water infiltrates from the environment into the product until an equilibrium state is reached [51]. This is associated with an increase in water activity and, consequently, the free water content increases. 

**Figure 2:**
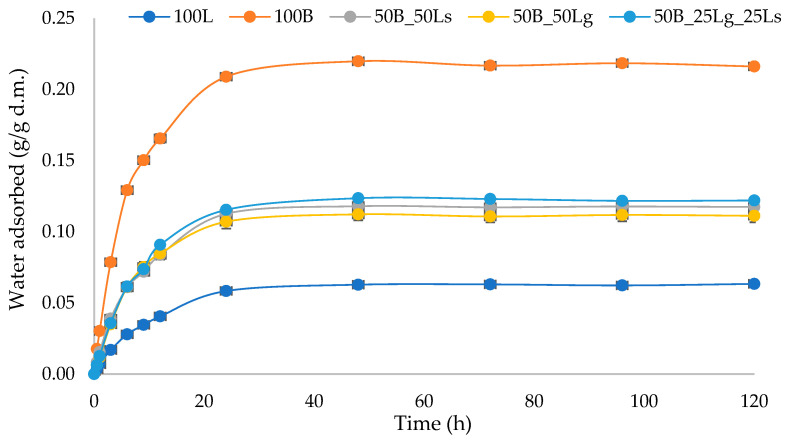
Kinetics of water vapor sorption of vegetable crispbreads based on beetroot (B) pomace and linseed (L).

The increasing presence of water allows microorganisms to grow or biochemical reactions to take place, and thus, product quality and shelf life are reduced [42]. Regarding the analyzed vegetable snack based on beetroot pomace and linseed, the most water was absorbed in the initial stage of sorption, and after 20 h, the system began to stabilize (Figure 2). The shape of the sorption curves is typical for food products with low water activities [36,52]. The sample containing beetroot pulp (100B) had the highest weight gain, and after 20 h, the water content of the material was 0.2 g/g d.m., while the sample containing linseed (100B) had the lowest weight gain, absorbing 0.05 g/g d.m. of water after 20 h. The addition of linseed to beetroot pomace resulted in a significant reduction in water adsorption, and values in the range of 0.10–0.12 g/g d.m. of water were obtained. The highest hygroscopicity (0.12 g/g d.m.) among the mixed samples was found for the sample with the mixture of whole and ground linseed (50B_25Lg_25Ls), while the lowest (0.10 g/g d.m.) was found for the of the sample with whole linseed (50B_50Ls). The lowest hygroscopicity may be due to the presence of whole seeds and their characteristics. In general, the seeds have a less developed sorption surface, and their seed coats have not been damaged in the crushing process, which may limit water vapor sorption. The high hygroscopicity of beetroot pulp snacks may be related to the high porosity of the raw material itself as well as the resulting snack. The beetroot pomace was used in the form of “chips”, which did not form a uniform or consistent consistency after baking. In the case of pure linseed snacks, their porosity may be reduced because of the mucilage formed, which binds the pulp together and reduces porosity. In addition, both raw materials used are a source of hygroscopic substances such as fiber or sugar, which also intensifies the ability of the tested snacks to absorb water [53]. Moreover, an increase in the humidity of the environment in which a product is stored causes a significant increase in water content and leads to adverse quality changes.

### 2.6. Water Vapor Sorption Isotherms

The sorption properties and water activity of food products are crucial in their production, storage, and transport processes. Hygroscopicity refers to the material’s ability to absorb water vapor in a humid environment and release water in a dry one, resulting in changes in the product’s water content [36]. Valuable information on this subject can be obtained through sorption isotherms, which determine the equilibrium relationship between the amount of water adsorbed per unit mass of the product and the water activity at constant total pressure and temperature [54].

**Figure 3:**
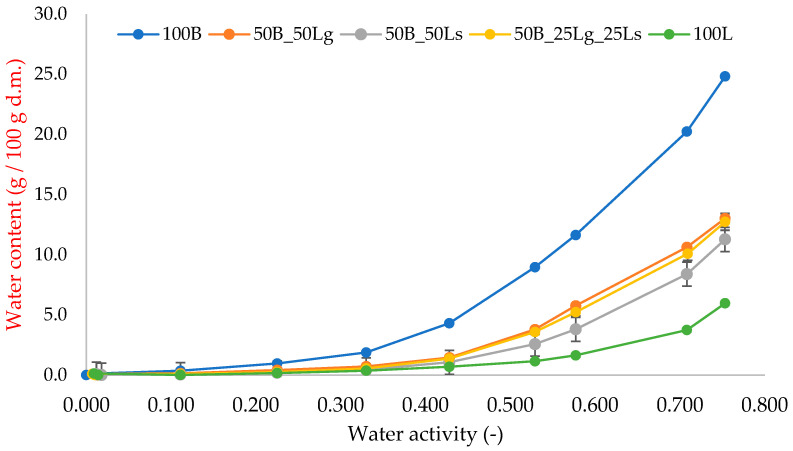
Water vapor sorption isotherms of vegetable crispbreads based on beetroot (B) pomace and linseed (L).

The water vapor sorption isotherms for the analyzed crispbreads based on beetroot pomace and linseed are presented in Figure 3. Both components have a high polysaccharide content, and the isotherm curves showed the shape typical for materials such as a type III according to the classification based on their shape and processes described by Brunauer et al. [55]. The greatest differences occurred at high water activity values (above 0.6), which may be related to the easier mobility of water molecules when the material becomes soft [56]. The sample of pure beetroot pomace (100B) had the highest weight change (25%), while the sample of pure linseed (100F) had the lowest (6%), which corresponds with the results of water vapor sorption kinetics (Figure 2). The addition of ground linseed (50B_50Lg) resulted in a higher weight change (13%) compared with the sample containing seeds (50B_50Ls), where the weight change was 11%. The addition of linseed to beetroot pomace resulted in a more than twofold decrease in weight changes. The higher mass change for sample 50B_50Lg may be related to the larger adsorption surface area that resulted from the grinding of the seed. The high mass change value for sample 100B may be due to the high porosity, which greatly facilitates water adsorption. The addition of linseed may result in reduced porosity and, therefore, less water is adsorbed. The other raw materials used, i.e., beetroot pomace and linseed, are a source of hygroscopic substances such as fiber and sugar, which also enhance the ability of the tested snacks to adsorb water [53].

### 2.7. Water Contact Angle

The wettability of the product surface was analyzed by observing the water contact angles between a water droplet and the vegetable crispbreads. Generally, small contact angles (equal to or below 90°) correspond to high wettability and characterize hydrophilic surfaces, while large contact angles (equal to or above 90°) indicate low wettability and the hydrophobic character of a material [57]. Regarding vegetable snacks, the wettability of the product is attributed to the sensory values, such as adhesives and texture, while eating and chewing. The results of the water contact angle of the vegetable crispbreads based on beetroot pomace and linseed are presented in Table 4. The analyzed samples showed an initial contact angle (0 s) between 78.66 ± 6.83° and 105.93 ± 4.5°, whereas the values for all samples containing beetroot pomace were above 90°, indicating the hydrophobic character of the surface. However, after 5 s of the analysis, the samples with a contact angle above 90° were those containing whole linseed and partly ground linseed, at 91.21 ± 3.54 and 98.05 ± 5.44, respectively. Nevertheless, all samples showed decreased values of the water contact angle at 5 s in comparison with the initial ones. Moreover, an increase in the time of the analysis was associated with a decrease in the contact angle until 0° at the time of 10 s, which is a relatively short time for complete water absorption. Statistically significant differences were observed among the samples containing beetroot pomace, linseed, and a combination of both components. 

The addition of linseed reduced the wetting angle, but the samples were still highly hydrophobic. This may be due to the decreased water sorption surface area that occurred as a result of the linseed and the greater uniform texture of the snack. The snacks obtained from ground linseed (50B_50Lg) showed a significantly lower value of the contact angle, of 92.10 at 0 s and 86.86° at 5 s, in comparison with semi-ground linseed (50B_25Lg_25Ls). This is probably due to the smoother surface when no seeds were present. In addition, these snacks were also thinner in comparison with the others. The overall high wetting angle of all snacks may be due to the presence of hydrophobic components such as oil and mucilage derived from linseed [58]. In addition, baking and hardening of the surface may have occurred during thermal processing, especially in the case of beetroot pomace, making the sorption of water droplets slower. The discrepancy in the results presented above may also be due to the different structures of the snacks, and the inconclusive results may be related to where the droplet was applied. The structure of the analyzed crispbreads is not uniform because of pores and seeds or the linseed mucilage present. This could be explained also by the surface roughness of the samples since there was a smoother surface on the support side of the crispbread because of the adhesion to the support during drying, which is a physical phenomenon connected with the formation of snack structure. The high hydrophobicity of the snacks allows the product to remain crunchy for longer and prevents softening. The snacks can be stored in dry and stable conditions without the risk of quality loss or mold formation. 

### 2.8. Texture

One important food quality parameter is texture, which can be analyzed using different methods. The breaking test that was used in this study corresponds to the breaking strength, which is generally the tensile or compressive load required to fracture or cause the sample to fail. It characterizes food texture and provides information on a product’s hardness or brittleness, thus indicating its quality. This method is usually used for food products [59]. Table 5 shows the results for texture obtained for the analyzed vegetable crispbreads. The range of values was between 7.80 and 18.86 N. Statistically significant differences were observed among the samples containing beetroot pomace, linseed, and a combination of both components (*p* < 0.05). The highest value during the breaking test (18.86 ± 1.77 N) was achieved by the pure linseed crispbreads (100L) and the lowest value (7.80 ± 2.03 N) by the pure beetroot crispbreads (100B). The softness of the 100B sample may be due to the high porosity of the snack (Figure 1) and the lack of gelling agent; thus, the structure was not well structured or compact. 

The addition of ground linseed (50B_50Lg) resulted in a significant increase in hardness (13.83 ± 1.08 N) compared with the values (8.08 ± 1.37) obtained for the sample containing whole linseed (50B_50Ls). This is probably attributed to the uniform distribution of raw components, their integrity, and the formation of continuous structures during drying. A similar tendency of higher texture values (12.61 ± 2.06 N) was observed for the snacks containing both whole and ground linseed (50B_25Lg_25Ls). The hardness of pure linseed crispbreads (100F) may be due to the high mucilage content derived from linseed. The mucilage binds the whole mass together and the structure becomes more compact. This may be also due to the greater surface area of the ground linseed and, therefore, the greater amount of mucilage. In addition, the ground linseed, because of its much smaller dimensions compared with the seeds, was distributed evenly throughout the mass and did not leave too much free space, resulting in a harder snack after baking. This is also connected with a higher thickness in comparison with the other samples because of the formation of compact structures during drying. The mucilage reduces the porosity of the snacks. Bashir et al. [60] prepared gluten-free cookies using linseed flour and showed that the hardness of the materials increased with an increasing concentration of linseed flour. The increasing hardness of the snacks may also have been influenced by the fiber present in linseed. Aydogdu et al. [61] studied the effect of adding fiber of different origins, including peas, oats, lemon, apples, and cakes. Their study showed that irrespective of the origin of the fiber, the firmness of the dough increased with increasing content of this ingredient, indicating that fiber content had a significant role in the formation of the product during baking.

### 2.9. Thermal Properties

**Figure 4:**
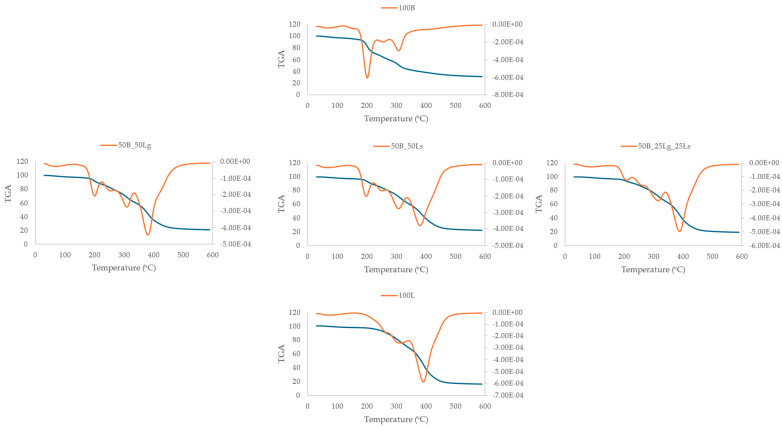
Thermogravimetric analysis (TGA) (blue) and derivative thermogravimetry (dTG) (orange) curves of vegetable crispbreads based on beetroot (B) pomace and linseed (L).

The first stage in thermogravimetric analysis (TGA), ranging from 25 to 200 °C, is attributed to the evaporation of water and volatile components [62]. The next stage, in the range of 200 to 350 °C, refers to the thermal decomposition of the components present in the snacks containing fat compounds or polysaccharides. The main transformations, associated with the loss of material mass, read above 300 °C, which can be due to the decomposition of the remaining organic components [63]. The results of thermogravimetric analysis (TGA) are presented in Figure 4 and Table 6.

In the first stage, the highest weight loss (3.16%) at the lowest temperature was achieved by the pure beetroot snacks (100B), which may indicate the low thermal stability of the product (Table 6). The other snacks obtained a higher temperature and lower weight. A similar observation was made for the second stage, in which this sample also achieved the highest weight loss (36.89%) as one, two, or three temperatures were noted. This is connected with the presence of oil in linseed since similar changes in the pure linseed snacks were observed. The thermogravimetric loss was calculated for all changes in this step and showed as one loss. The addition of beetroot pomace to the linseed caused an increase in the weight loss of the vegetable snacks. The higher weight loss caused by the presence of beetroot pomace may be due to the higher water activity compared with the other samples and may be related to the degradation of some of the beetroot components that have a low molecular weight and low glass transition temperatures, such as sucrose, fructose, glucose, and acids [64]. In this temperature range, the snacks lost their thermal stability, as shown in the graph (Figure 4). In the third stage, weight loss was at a similar level in the range of 16.33–18.60%. 

The addition of linseed increased the weight loss. During the second and third stages, degradation of fatty compounds and polysaccharides may have occurred. The fourth stage was characterized by the highest weight loss for the linseed variants. At temperatures above 350 °C, there was a degradation of the remaining organic components found in rich quantities in linseed. The fifth stage recorded the lowest weight loss from 1.25% for pure linseed snacks to 1.95% for pure beetroot snacks.

### 2.10. Fourier Transform Infrared Spectroscopy (FTIR)

**Figure 5:**
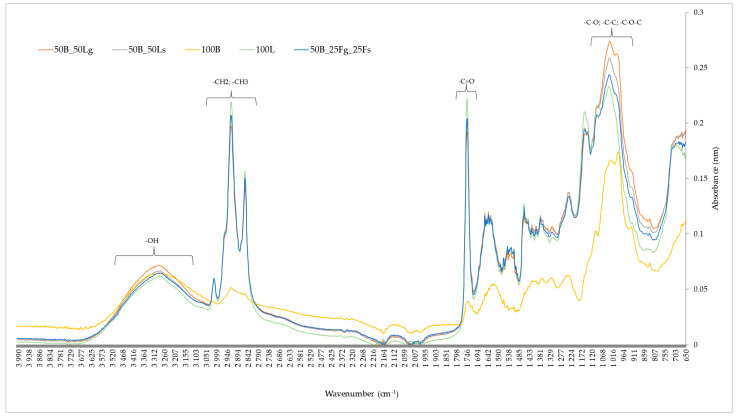
The Fourier transform infrared spectroscopy (FT-IR) spectra of vegetable crispbreads based on beetroot (B) and linseed (L).

The presence of functional groups in the material related to the chemical composition, such as carboxylic acid presence, was investigated using Fourier transform infrared spectroscopy (FTIR). This analysis identifies specific wavelengths that are assigned to different functional groups, and the results are presented in Figure 5. Specific wavelengths are assigned to different functional groups. In the range from 3600 to 3000 cm^−1^, the -OH group stands out, which is characteristic of alcohols, for example. The bands in the range of 2950–2750 cm^−1^ correspond to the presence of -CH2 and -CH3 alkyl groups, and the band in the range of 1750–1650 cm^−1^ corresponds to a carbonyl group, which are characteristic of, for example, aldehydes. At 1150–900 cm^−1^, the presence of groups such as C-O, C-C, and C-O-C can be noted, which are found in saccharides, e.g., glucose [65].

For the analyzed vegetable crispbreads, the first peak was noticed in the 3500–3000 cm^−1^ range, indicating the presence of the -OH group. The highest absorbance (0.07 nm) was observed for the snack consisting of beetroot pomace and ground linseed (50B_50Lg). This may be due to the formation of high water activity in the beet pulp and the large amount of mucilage that was produced by the addition of water. The other samples had similar absorbance values. The absorbance value of the -OH group, however, is not high compared with the other functional groups, which may be due to the evaporation of a significant amount of water during baking. A large peak appeared in the wavelength range of 2950–2700 cm^−1^, which may indicate the presence of alkyl groups. The spectra show that the snack containing pure linseed (100L) had the highest presence of these functional groups, thus increasing the nutritional value of the snack. The absorbance for this snack was 0.22 nm. The lowest absorbance, on the other hand, was characterized for the pure beetroot snacks (100B), and its value was 0.05 nm. This may be due to the use of pomace and heat treatment, which may have degraded the alkyl compounds. Another large peak in the range of 1750–1650 cm^−1^ indicates the presence of a carbonyl group, which is present in aldehydes, esters, and carboxylic acids.

The snacks containing pure linseed are carriers of the carbonyl group because of their high fatty acid content and absorbance of 0.22 nm. In the case of beet pomace, this value is only 0.04. The addition of linseed to pomace significantly affected the presence of these groups. High absorbance values were also reached by peaks in the range of 1150–900 cm^−1^, indicating the presence of groups such as C-O, C-C, and C-O-C. All the snacks with linseed addition achieved significantly higher absorbance values compared with the pure beetroot (100B) crispbreads. The addition of linseed significantly increased the content of these functional groups. Aztatzi-Rugerio et al. [66] mentioned that temperature-sensitive compounds, such as betalains, lose stability during heat treatment, causing structural changes in the molecules. The last spectral region below 900 cm^−1^ is responsible for crystalline regions and indicates conformational changes in test materials [67]. Kushwaha et al. [13] also noted the presence of -OH, -CH, -NH, and -CO groups during FTIR analysis of dried beet pulp. Linseed is an oilseed crop. Suri et al. [68] performed FTIR analysis of linseed oil at 25 °C. The highest peaks were obtained at 2929, 1745, 1161, and 725 cm^−1^, among others. At similar wavelengths, the highest absorbance was also obtained for the pure linseed snacks (100L), confirming that this component is a carrier of important chemical compounds.

### 2.11. Betalain Content

Betalains are the pigments that give beetroot its red and yellow color. In addition to providing color, they exhibit antioxidant or antimicrobial properties. Despite their beneficial properties, betalains are sensitive to external factors, e.g., temperature, which can pose a problem during processing [69]. Therefore, apart from the vegetable snacks, betalains were also evaluated in the fresh beetroot pomace to show the effect of the drying process on this bioactive compound. The results of the betalain content are presented in Table 7 Raw beetroot pomace had the highest red and yellow pigment content at 241.14 ± 25.96 mg betanin/100 g d.m. and 121.63 ± 8.23 vulgaxanthin/100 g d.m., respectively. After the heat treatment of the beetroot pomace (160 °C, 50 min), the content of betalains decreased significantly and was in the range of 3.87–51.44 mg/100 g d.m. for red pigments and 12.29–171.12 vulgaxanthin/100 g d.m. for yellow pigments. This decrease in the values indicates their sensitivity to high-temperature treatment. Statistically significant differences were observed among samples containing beetroot pomace, linseed, and a combination of both components (*p* < 0.05). The highest red and yellow pigment content, at 51.44 ± 2.77 mg betanin/100 g d.m. and 171. 13 ± 7.16 mg vulgaxanthin/100 g d.m., respectively, was characterized by the pure beetroot crispbreads (100B), while the lowest values (3.87 ± 1.65 mg betanin/100 g d.m. and 12.29 ± 3.59 mg vulgaxanthin/100 g d.m.) were observed for the pure linseed crispbreads (100L). The addition of linseed had a reducing effect on the content of betalains in the snacks as it is not a carrier of these pigments. 

The content of red and yellow pigments in the mixed beetroot pomace and linseed crispbreads (50B_50Lg, 50B_50Ls, and 50B_25Lg_25Ls) were of a similar range, from 14.59 ± 1.38 to 16.62 ± 1.94 mg betanin/100 g d.m. for red pigment content and from 50.02 ± 3.59 to 51.31 ± 0.45 mg vulgaxanthin/100 g d.m.) for yellow pigment content. The results were not statistically significant (*p* < 0.05). This observation confirmed that the form in which the linseed was added does not affect the content of betalains. There was a greater reduction in red pigments in the snacks in comparison with yellow pigments. Kidoń and Czapski [70] studied the effect of heat treatment on betalain pigment content. One hour of heating at 90 °C did not affect changes in the tested parameters of the dried beetroot, whereas three hours of exposure to high temperature resulted in a degradation of the red betalain pigments by about 75%. Red pigments degrade significantly faster following heat treatment compared with yellow betaxanthins [37]. Vulić et al. [71] studied the content of betalains in beetroot pomace extracts of different varieties. For red pigments, the results ranged from 0.038 to 0.368 mg betanin/100 g d.m. and for yellow pigments from 0.003 to 0.004 mg vulgaxanthin/100 g d.m., depending on the variety tested. The authors explained that the differences in values might be due to the variety used or the growing conditions. Juice production generates pomace as a waste product, which, regardless of the form (pomace or extract), is a source of many valuable components such as betalains and fiber. Therefore, it is worthwhile to manage and use it to enrich food products.

### 2.12. Content of Bioactive Compounds and Antioxidant Activity

Polyphenols are some of the most important ingredients in the human diet because they carry various health benefits through the suppression of oxidative stress. The polyphenol content of food depends on temperature, pH, and the plant part used, among other factors [72]. The results for the content of total polyphenols were between 730 and 948 mg chlorogenic acid/100 g d.m. and are presented in Table 8. The sample prepared from pure linseed (100L) showed the highest polyphenol content, indicating that this snack component has a very strong antioxidant system, being particularly rich in lignans. In addition to lignans, linseed contains large amounts of phenolic compounds such as ferulic acid, ferulic acid, cinnamic acid, vanillic acid, *p*-coumaric acid, and gallic acid [73]. The snacks consisting of pure beetroot pomace compared with pure linseed had a lower polyphenol content, which may be due to the use of pomace. The polyphenol content of the linseed and beetroot pomace crispbreads ranged from 730 to 830 mg chlorogenic acid/100 g d.m. Statistically significant differences were observed between the samples containing pure linseed and the samples containing beetroot pomace. The pure linseed crispbreads (100L) had the highest polyphenol content of the control samples (948 ± 72 mg chlorogenic acid/100 g d.m.), while the pure beetroot crispbreads sample (100B) had the lowest value (820 ± 27 mg chlorogenic acid/100 g d.m.). 

Among the mixed snacks, the samples containing whole linseed (50B_50Ls) had the highest polyphenol content (830 ± 17 mg chlorogenic acid/100 g d.m.), which may have been due to the use of seeds, where their closed form protected the bioactive compounds from degradation during thermal processing. On the other hand, the samples containing ground linseed (50B_50Lg) had the lowest polyphenol content (730 ± 26 mg chlorogenic acid/100 g d.m.). This may be due to the high exposure to temperature because of the milling of the seeds. The resulting snacks are also characterized by low water content and, consequently, high dry matter (Table 2). Polyphenols are water-soluble and may have been lost with water by evaporation [74]. In addition, Orhan et al. [75] suggested that genotype, location, and cultivation techniques, as well as differences in plant maturity, affect phenolic content. Also, external factors such as light, temperature, and the presence of nutrients in the soil can affect polyphenol content in raw materials. Malakar et al. [74] studied the effect of heat treatment on the polyphenol content of beetroot. Their study showed that as drying time and temperature increased, the total polyphenol content decreased.

The determination of the oxidative activity of the product is very complex; thus, no single method is capable of entirely depicting natural reactions occurring in vivo. Chemical-based analyses can be further subdivided into different methods for assessing antioxidant activity. Among them are those based on the scavenging activity toward a stable free radical (2,2-diphenyl-1-picrylhydrazyl (DPPH) and 2,2′-azino-bis(3-ethylbenzothiazoline-6-sulfonic acid) (ABTS)) or the reduction of metal ions (ferric ion reducing antioxidant potential (FRAP) or reducing power). These analyses allow for the determination of the potential health benefits of bioactive substances and their effect on neutralizing harmful free radicals in the body [76]. Both ABTS and DPPH, as well as reducing power, were used in this study to evaluate the antioxidant activity of the vegetable crispbreads based on beetroot pomace and linseed. The results are presented in Table 9. 

The free radical quenching capacity of ABTS in the tested snacks was in the range of 1.46–2.58 mg Trolox/g d.m. Statistically significant differences were observed between the snacks containing pure beetroot pomace and the other samples with the addition of linseed. The beetroot pomace snack (100B) had the highest free radical quenching capacity of ABTS (2.58 mg Trolox/g d.m.), while the lowest value (1.46 mg Trolox/g d.m.) was shown for the snacks containing ground linseed (50B_50Lg). On the other hand, when whole linseed was used (sample 50B_50Ls), a higher quenching capacity was observed (1.82 mg Trolox/g d.m.), which may be due to the better preservation of bioactive compounds in linseed in the natural form without milling. The low ABTS values may be due to the mixture with beetroot pomace and its interactions, which has been stripped of its juice and thus many of its bioactive compounds, and the use of high temperatures during heat treatment. During heat treatment, the degradation of betalains can lead to the formation of other phenolic compounds, increasing antioxidant activity [77]. Therefore, lower ABTS values may be connected with the lower betalain content of the analyzed vegetable snacks, as shown in Table 7.

The DPPH assay tests antioxidant activity by measuring the absorbance of residual radicals in the reaction medium. DPPH measurement is useful to determine the ability of substances to neutralize free radicals that are associated with oxidative stress [78]. The antioxidant activity of the analyzed vegetable crispbreads ranged from 2.20 to 2.48 mg Trolox/g d.m. (Table 8). For the control sample, the beetroot pomace snack (100B) had the highest antioxidant activity (2.41 ± 0.02 mg Trolox/g d.m.), while the lowest value (2.20 mg Trolox/g d.m.) was shown for the pure linseed snack (100F). For the mixed snacks, the addition of linseed did not result in statistically significant differences (*p* < 0.05). Raczyk et al. [79] studied the effect of the addition of beetroot, carrot, and tomato juice to wheat bread on antioxidant activity. Compared with the other vegetables, beetroot had the highest antioxidant value, as confirmed by both ABTS and DPPH analyses. The use of beetroot pomace in the analyzed vegetable snacks, in addition to waste management as a circular approach, allows for the raw material with the highest potential antioxidant activity to be carried out. Similar observations were noted by Gramza-Michałowska and Człapka-Matyasik [80] for carrot chip extract. The authors studied the scavenging activity of the DPPH radical, which was 3.47 mg Trolox/g d.m., and described this antioxidant efficiency as a medium. For ABTS, the value was 2.23 mg Trolox/g d.w., and the antioxidant efficiency was described as low.

The reducing capacity of a compound can be used as an indicator of the potential antioxidant activity of many products [81]. The iron-reducing power of the analyzed vegetable crispbreads ranged between 7.90 and 10.00 mg Trolox/g d.m. (Table 9). The sample prepared from pure linseed (100L) had the highest value (10.00 ± 0.29 mg Trolox/g d.m.), while the lowest value (7.80 ± 0.16 mg Trolox/100 d.m.) was observed for the snacks containing ground linseed (50B_50Lg). Statistically significant differences were observed among the samples containing beetroot pomace, linseed, and a combination of both components (*p* < 0.05). The low value of the reducing capacity (7.90 ± 0.1 mg Trolox/g d.m.) of pure beetroot snacks (100B) may be due to the use of beet pomace, which was stripped of its juice and subjected to high temperatures that may have led to the degradation of thermolabile components, which are antioxidants. On the other hand, the high value of the pure (100L) or linseed-containing crispbreads is due to their natures, being carriers of many antioxidants, including polyphenols. The results were also influenced by the form of linseed used, and for snacks containing ground linseed (50B_50Lg), the values were 7.80 ± 0.16 mg Trolox/g d.m, which were significantly lower in comparison with the sample containing whole linseed (50B_50Ls), which had values of 8.94 ± 0.35 mg Trolox/g d.m. This may be also attributed to the higher heat exposure because of the fragmentation of linseed. Moreover, Alachaher et al. [81] mentioned that the high antioxidant activity of linseed may be due to the presence of polyphenols, which act as reducing agents. For the analyzed crispbreads, the pure linseed snacks (100F) showed the highest value of total polyphenols (Table 8) and thus had the highest reducing power. The addition of linseed had a positive effect on the antioxidant capacity of the analyzed vegetable crispbreads.

### 2.13. Sensory Analysis

**Figure 6:**
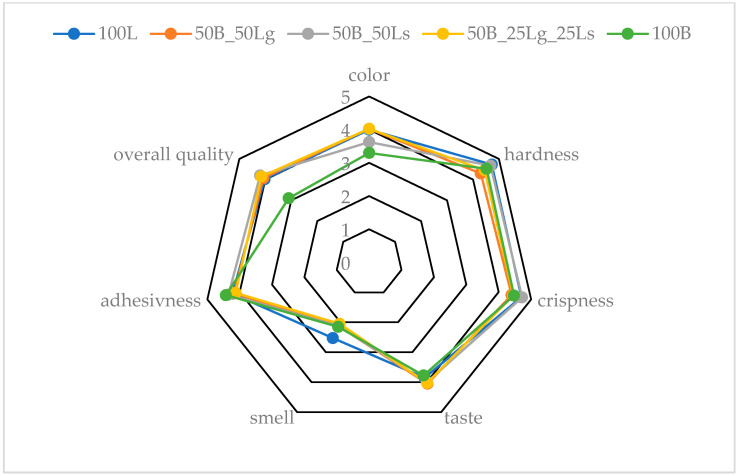
Sensory evaluation of vegetable crispbreads based on beetroot (B) pomace and linseed (L).

During the sensory evaluation, the color, hardness, crunchiness, taste, aroma, adhesiveness, and overall quality of the vegetable crispbreads were assessed (Figure 6). 

During the evaluation, consumers chose the most favorable color of the snack. If the color was satisfactory, a score of 5 was given, and if the color was uninviting, the consumers gave a score of 1. If the sample offered a lot of resistance when biting, hardness was scored 5, and if the sample was soft and offered no resistance, a score of 1 was given. The same applied to crunchiness. If there was a lot of crunchiness when biting, a score of 5 was given. If no crunchiness was perceived, the consumers gave a score of 1. Taste is a subjective matter, but the tastier the flavor, the higher the score. If an intense odor was perceived by the consumer, a score of 5 was given. If the odor was not perceptible, a score of 1 was given. For adhesiveness, the sensation of the sample “sticking” to the teeth and being difficult to remove was determined. If this was evident, the consumers awarded the sample a score of 5, if they did not feel this, a score of 1 was given. The overall score referred to the overall sensory experience of the consumers. If the overall rating was very good, a 5 was awarded, if it was bad, a 1 was given. 

The snacks containing ground (50B_50Lg) and the mixture of ground and whole linseed (50B_25Lg_25Ls) had the most desirable color, while the sample containing 100% beetroot pomace (100B) had the lowest. This may have been because the ground linseed used turned lighter in color, making it more desirable to the consumer (Table 1). The snacks containing pure linseed (100L) and whole linseed combined with beetroot pomace (50B_50Ls) appeared to be the hardest and crispest. This may have been due to the presence of whole seeds and mucilage production and thus, the formation of the structure during drying. The snacks containing ground linseed (50B_50Lg) had the best taste, whereas the worst-rated sample from a taste point of view was the pure beetroot snack (100B). The sensory evaluation showed that the addition of linseed had a positive effect on the taste of vegetable crispbreads. The smell of the snacks was not intense and was at a similar level for each sample. Adhesiveness was also assessed at a similar level. The results for crispness, flavor, odor, and adhesiveness were not statistically different. The vegetable snacks prepared from beetroot pomace combined with whole linseed (50B_50Ls) had the best overall quality, while the pure beetroot snacks (100B) had the weakest. This shows that the addition of linseed affected the positive consumer perception.

## 3. Materials and Methods

### 3.1. Materials

Beetroot (*Beta vulgaris* L.) was purchased from a local supermarket in Warsaw, Poland, and stored at 4 °C before experiments. Golden linseed (*Lini semen*) (Oleofarm, Wrocław, Poland) was purchased from a local shop and stored at room temperature before experiments. All reagents were of analytical grade and were purchased from Chempur (Piekary Śląskie, Poland). The following reagents were used for the analytical determination: phosphate buffer (pH 6.5), 80% ethyl alcohol, Folin Ciocalteau reagent, saturated sodium carbonate, ethanol extract, 2,2-azynobis(3-ethylbenzothiazoline-6-sulfonate) cation radical, potassium persulfate, 2,2-diphenyl-1-picrylhydrazyl, 99% methanol solution, 1% aqueous solution of potassium cyanogelazat, 10% trichloroacetic acid, and 0.1% iron (III) chloride solution and distilled water.

### 3.2. Production of Vegetable Snacks

The beetroots were washed with tap water, dried in the air, and peeled. Then, the vegetables were cut and used for juice-pressing. Beetroot pomace was obtained after a juice preparation using a juicer (Zelmer, Warsaw, Poland). The resulting pomace had 12.11 ± 0.29% dry matter, as evaluated by the drying method at 105 °C for 24 h. Linseed was used whole or ground using a grinder (BOSCH, Stuttgart, Germany) and as a combination of both forms in the proportion 50:50. The linseed was mixed with boiling water in a ratio of 2:1 and left for 5 min until the mass gelled. Three variants were produced, consisting of 50% pomace and 50% linseed (50B_50Ls), 50% pomace and 50% ground linseed (50B_50Lg), and 50% pomace with the 25% ground linseed and 25% whole linseed (50B_25Ls_25Lg). Two control versions were created, one of which consisted of 100% beetroot pomace (100B) and the other of 50% whole seeds and 50% ground seeds (100L). Salt was added to each variant (0.5 per 100 g of fresh mass). Each variant was spread on 30 × 40 cm baking sheets using a layer applicator model Proceq Zehntner ZAA2300 (Screening Eagle Technologies AG, Zurich, Switzerland) with a constant thickness of fresh mass of 5 ± 1 µm, resulting in a constant snack thickness after drying among the samples. The snacks were baked in a Picollo oven (Wachtel Polska Sp. z o.o., Wroclaw, Poland) for 50 min at 160 °C. After baking, the snacks were cooled to room temperature and packed in low-barrier PET12/Al8mat/PE80 film bags (Pakmar Sp. z o.o., Garwolin, Poland), which were sealed using a PFS/FS 300 C laboratory impulse sealer (Neopack Sp. z o.o., Warsaw, Poland).

### 3.3. Observation of the Surface Structure and Cross-Sections of the Crispbreads

Samples measuring 50 × 50 mm were placed on a measuring table and were observed using a microscope VHX 970F (Keyence International, Mechelen, Belgium). A Z20 objective and ×50 magnification were used. Observations of the cross-sectional structure and surface of the snacks were made with a Quanta 200 scanning electron microscope (FEI Company, Fremont, CA, USA). Snacks measuring 10 × 10 mm were placed on a measuring table using a 9 mm diameter PELCO carbon paste (Pik Instruments Sp. z o.o., Piaseczno, Poland). The test was carried out in a low vacuum of 0.35–1 torr at 250× magnification.

### 3.4. Color

Color measurement was evaluated in 10 replicates using a Minolta colorimeter (model CR-400, Konica Minolta, Tokyo, Japan) in the CIE L*a*b* system, where L* is lightness and a* and b* are trichromatic coordinates. The reference material was a standard white plate with constant color parameters.

### 3.5. Dry Matter Content

The determination of the dry matter content of the snacks was carried out using the drying method and was performed in triplicate. Samples were ground with an analytical mill A 11 basic (IKA Poland Sp. z o.o., Warsaw, Poland), weighed (3 ± 0.0001 g) into a vessel, and then dried at 105 °C in a laboratory dryer (SUP 65 WG, WAMED, Warsaw, Poland) for 24 h. The dry matter content was calculated based on the sample mass before and after drying and expressed in %.

### 3.6. Water Activity

Water activity was tested in the raw mass and after baking using an AquaLab Series 3TE apparatus (Decagon Devices, Pullman, WA, USA). The determination was performed with an accuracy of ±0.001. Measurements were performed at 25 °C in triplicate.

### 3.7. Water Vapor Adsorption Kinetics

A saturated solution of sodium chloride (NaCl) was used to achieve constant ambient relative humidity (75%) at 25 °C. Water vapor sorption kinetics were measured after 0.5, 1, 3, 6, 9, 12, 24, 48, 72, 96, and 120 h. Samples of 250 ± 5 mg were cut into small pieces and weighed periodically. The measurement was carried out at 25 ± 1 °C, and at least three replicates were performed for each type of snack. The kinetics of water vapor adsorption was described by the classical diffusion equation based on Fick’s law through an L-thick membrane [82]:

(1) $$\frac{M_{t}}{M_{\infty }}=1-\sum_{n=0}^{\infty }\frac{8}{(2+1{)}^{2}\pi^{2}}\exp\left[\frac{-D(2\pi +1{)}^{2}\pi^{2}t}{4L^{2}}\right]$$

where *t* is time (s) and *M_t_*/*M*_∞_ is the total amount of water vapor that was adsorbed by the sample at time *t*. The measurement was performed in 3 replicates.

### 3.8. Water Vapor Adsorption Isotherms

Isotherms of water adsorption by the snacks were determined using an AQUADYNE DVS-2HT dynamic water vapor sorption analyzer (Quantachrome Instruments by Anton Paar Sp. z o.o., Warsaw, Poland). Crispbread samples weighing 20 ± 1 mg were placed in a glass pan and subjected to a series of relative humidity (RH) values (0, 10, 20, 30, 40, 50, 60, and 75%) until the sample reached equilibrium at 25 °C. The equilibrium criterion at each relative humidity was a percentage rate of mass change over time (d_m_/d_t_) ≤ 0.002% min^−1^ in 10 min. The experimental data points were analyzed using Microsoft Excel 2019 with DVS Standard Analysis software. The data were processed using OriginPro 8.0 software (OriginLab Corporation, Northampton, MA, USA). The measurement was performed in 2 replicates.

### 3.9. Water Contact Angle

The wettability angle analysis was carried out using an OCA 25 goniometer (DataPhysics Instruments, Filderstadt, Germany). Snacks measuring 1 × 1 cm were used for the measurement. A syringe was filled with distilled water and mounted in a holder over the material. A needle was placed in the correct position to obtain a sharp image of the needle and surface. A 10 μL droplet of distilled water was produced using a micrometer screw. The shape of the droplet was recorded by a digital camera and transferred to a computer using SCA20_U software. The image was magnified, making it possible to determine the angles between the material and the contacting water droplet after 0, 5 and 10 s. The absorption time of the droplet was also measured. The measurement was performed in a minimum of 6 repetitions.

### 3.10. Texture

The texture snacks of 5 × 5 cm were examined by the breaking test at 1 mm/s using a TA-XT2i texturometer (Stable Micro Systems, Surrey, UK) as the max. the force needed to break the sample. The results obtained from the measurements were collected using Texture Expert software (latest version 2.64). The measurement was performed in 6 repetitions.

### 3.11. Thermal Properties

Thermogravimetric analyses were performed using a TGA thermal analyzer (Mettler Toledo, Warsaw, Poland) to determine the thermal stability and degradation of the samples. Each snack sample (5 mg) was heated at 5 °C min^−1^ from 30 to 600 °C in a nitrogen atmosphere (the N_2_ flow rate was 50 m min^−1^). The measurement was performed in 1 replicate. TGA and dTG curves were obtained from the differential TGA values. 

### 3.12. Fourier Transform Infrared Spectroscopy

Fourie transform infrared spectra were determined by the total attenuated reflectance (ATR) technique using a model Cary-630 spectrometer (Agilent Technologies, Cary, NC, USA). The spectra of the analyzed samples were recorded in the absorption range of 4000–650 cm^−1^ with a resolution of 4 cm^−1^. Each spectrum was the average of 32 interferograms and was presented as the dependence of absorbance on wave number. The measurement was performed in 3 replicates.

### 3.13. Betalain Contents

The analysis of betalains was carried out in triplicate using the spectrophotometric method described by Janiszewska and Włodarczyk [83] with some modifications. Samples were ground using a laboratory grinder A 11 basic (IKA Poland Sp. z o.o., Warsaw, Poland). The pigments were extracted from the sample with phosphate buffer (pH 6.5). Samples of 0.3 g each were weighed, and 50 mL of phosphate buffer was added. The falcons were placed in a shaker for 10 min at 6000 rpm. The prepared extract was transferred to a centrifuge model 4K15 (Polygen Sp. z o.o., Wrocław, Poland) for centrifugation at 15,000 rpm for 5 min. The absorbance of the solution was measured at wavelengths of 476, 538, and 600 nm using a Helios Gamma spectrophotometer (Thermo Spectronic, Cambridge, UK). A buffer solution was used as a standard. Measurements were taken no later than 20 min after dye extraction. The determination of betalains, including red and yellow pigments, was calculated for betanin (mg betanin/100 g d.m.) and vulgaxanthin-I (mg vulgaxanthin-I/100 g d.m.), respectively. The calculation of pigment content was based on A1% absorbance values, which are 1120 for betanin (at 538 nm) and 750 for vulgaxanthin-I (at 476 nm). According to the methodology, the absorbance at 600 nm was measured and used to correct for the amount of impurities. For red pigment, the following calculation equation was used, which considers the presence of impurities (A_600_):

Red = (R·1.095(A_538_ − A_600_))/(d.m.·1120) (2)

For yellow pigments, the following calculation equation was used, which considers the absorbance of red pigments:

Yellow = (R·(A_476_ − A_538_ + 0.677·1.095(A_538_ − A_600_)))/(d.m.·750) (3)

where R—dilution of the sample (5000 mg); A_476_, A_538_, and A_600_—absorbances; m— mass of the sample (g); d.m.—dry matter (g/g); and 1.095—correction factor for the increase in absorbance due to the presence of impurities at 576 nm by the presence of impurities at 538 nm; 1120—absorbance of a 1% betanin solution determined at 538 nm in a 1 cm diameter cuvette; 0.677—correction factor for the increase in absorbance due to the presence of impurities at 538 nm; 0.677—correction factor for the increase in absorbance due to the presence of impurities at 476 nm; and 750—absorbance of a 1% volgaxanthin solution determined at 476 nm in a 1 cm cuvette.

### 3.14. Determination of Bioactive Compounds

#### 3.14.1. Preparation of Material

The baked and cooled snacks were ground in an analytical mill A 11 basic (IKA Poland Sp. z o.o., Warsaw, Poland). Approx. 0.3 g of ground material was weighed on an analytical balance to the nearest 0.0001 g into a 15 mL plastic falcon and mixed with 10 mL of extraction reagent (80% ethyl alcohol). Extraction was carried out on a shaker Multi Reax (Heidolph Instruments GmbH & Co., Schwabach, Germany) for 24 h at 6000 rpm at room temperature. Samples were centrifuged for 2 min at 5000 rpm using a laboratory centrifuge MegaStar 600 (VWR International Sp. z o.o., Gdańsk, Poland). The supernatant was transferred to resealable 0.2 mL tubes. Three extracts were made for each sample.

#### 3.14.2. Determination of Total Polyphenols

The spectroscopic method with Folin Ciocalteau reagent was used for the determination of total polyphenols. Ethanol extracts of 10 µL and distilled water of 10 µL were applied to plastic 96-well plates. To the resulting solution, 40 µL of 5-fold diluted Folin Ciocalteau reagent was added and mixed; then, after 3 min, 250 µL of saturated sodium carbonate was added. Everything was mixed thoroughly. A light-free incubation at room temperature was carried out for 60 min. Absorbances were measured using a plate reader Multiskan Sky (Thermo Electron Co., St. Louis, MO, USA) at 750 nm. The blank was prepared analogously, and the extract was replaced with an extraction reagent. Determinations were performed in 3 replicates for each extract. The content of phenolic compounds was calculated using a calibration curve prepared for chlorogenic acid (Sigma Aldrich, Buchs, Switzerland) in the range of 0–100 µg/mL (R^2^ = 0.998). The following formula from the standard curve was used for the calculation: y = 512.77x − 0.9798.

#### 3.14.3. Determination of Antioxidant Activity against the ABTS Radical

To determine antioxidant activity, a spectrophotometric method was used to determine the Fe^3+^ ion-reducing ability of the cation radical 2,2-Azinobis(3ethylbenzothiazoline-6-sulfonate). A starting solution (ABTS+) was prepared. In a 10 mL volumetric flask, 0.0384 g of 2,2-azynobis(3-ethylbenzothiazoline-6-sulfonate) cation radical was weighed on an analytical balance to the nearest ±0.0001 g, and then 0.0066 g of potassium persulfate was added and made up to 10 mL with distilled water. The prepared solution was mixed thoroughly and placed in the refrigerator for 12 h. To the 96-well plates, 10 µL each of extract and 250 μL of radical solution were added. Everything was mixed, and after 6 min, the absorbance was measured at 734 nm wavelength. The antiradical activity was determined from the decrease in the absorbance of the radical solution in the presence of the antioxidant and expressed as mg Trolox/g raw material. The measurement was performed in 3 replicates.

#### 3.14.4. Determination of Antioxidant Activity against the DPPH• Radical

To determine antioxidant activity, a spectrophotometric method was used to determine the Fe^3+^ ion-reducing ability of the 2,2-diphenyl-1-picrylhydrazyl (DPPH•) radical. The determination began with the preparation of a starting solution of DPPH•. Into a 100 mL volumetric flask, 0.025 g of 2,2-diphenyl-1-picrylhydrazyl radical was weighed on an analytical balance with an accuracy of ±0.0001 g and made up to the final volume with a 99% methanol solution. The solution was stored in a dark place at 4 °C for a minimum of 24 h to thoroughly dissolve the radical. The analyte solution was diluted 10-fold. Then, 10 μL of extract and 250 µL of the radical solution were each added to 96-well plates. Everything was mixed, and after 10 min, the absorbance was measured at 515 nm. The antiradical activity was determined from the decrease in the absorbance of the radical solution in the presence of the antioxidant and expressed as mg Trolox/g raw material. The measurement was performed in 3 replicates.

#### 3.14.5. Reduction Power of Iron Ions

A total of 25 µL of extract, 75 µL of distilled water, and 50 µL of 1% aqueous solution of potassium cyanogelazate were pipetted and mixed. The mixture was placed at 50 °C in an incubator INCU-Line ILS 10 (VWR International Sp. z o.o., Gdańsk, Poland). After 20 min, it was removed from the incubator to add 50 µL of 10% trichloroacetic acid. Then, 100 µL of the resulting solution was taken, and 100 µL of distilled water and 20 µL of 0.1% iron (III) chloride solution were added and mixed. After 10 min, the absorbance value of the solutions at 700 nm against the reagent sample was measured using a plate reader. The value of the reducing power of iron ions for each sample was expressed in terms of mg of Trolox. The measurement was performed in 3 replicates.

### 3.15. Sensory Evaluation

A sensory evaluation of the snacks was conducted using a 5-point rating scale by 40 people. The evaluators were students and employees of the Institute of Food Science from the age group of 20–45 years. The quality attributes evaluated were taste, aroma, color, firmness, crunchiness, adhesiveness, and overall acceptance of the products.

### 3.16. Statistical Analysis

Statistical analysis was carried out using Statistica 13 software (StatSoft Inc., Tulsa, OK, USA). One-way analysis of variance (ANOVA) with Tukey’s post hoc test was performed to detect significant differences in the properties of the samples at the 0.05 significance level.

## 4. Conclusions

The use of beet pomace and golden linseed makes it possible to obtain natural gluten-free vegetable snacks in the form of crispbreads with beneficial physicochemical and sensory properties. This is a good example of a circular approach in which beetroot pomace is used as a waste material from the food industry in the development of a new product, which is a source of bioactive ingredients such as polyphenols and betalains. Beet pomace is a good source of red and yellow pigments, thus enriching the snacks with these pigments. The results of Fourier transform infrared spectroscopy showed the presence of functional groups such as -OH, -CH2, -CH3, -C=O, -C-O, -C-C, and -C-O-C in the tested snacks, indicating that developed vegetable crispbreads are a source of alkyl or carbonyl groups. The addition of linseed positively shaped the physical characteristics of vegetable snacks, such as texture, and provided bioactive components. In addition, it had a positive effect on the color of the crispbreads by providing a lighter color toward yellow and increasing the hardness shaping their texture. The heat treatment used ensured that the water activity and water content of all snacks were low, thus increasing microbiological safety. The high hydrophobicity ensured the crispness of the crispbreads. Because of the presence of hydrophilic compounds in both beetroot pomace and linseed, it is important to store crispbreads in a low-humidity environment. This will limit the occurrence of adverse quality changes. 

Among all the analyzed vegetable crispbreads, the snacks consisting of beetroot pomace and whole linseed (50B_50Ls) had the highest content of polyphenols and the highest antioxidant capacity. The best overall quality of this snack may be due to the high hardness and crispness of the snack. In addition, it may be a result of how crispbread-type snacks are associated—crisp and with a high but acceptable hardness. The use of unground linseed ensured that as many antioxidant compounds as possible were retained. The presence of antioxidants combats free radicals in the body, which can negatively affect human health. This variant showed the best overall quality during the sensory evaluation. Therefore, vegetable crispbreads made from beetroot pomace and linseed can be used as a daily snack for meals such as a replacement of bread or different classic crisps or salty sticks. The use of the chosen raw materials increases the nutritional value of the snacks and provides valuable bioactive ingredients such as antioxidants. Moreover, this is a safe option for people with coeliac disease, as the snacks are gluten-free. A positive aspect of the analyzed vegetable snacks is also the use of waste material, which is in line with the idea of “less waste” and the use of linseed as a gelling agent; therefore, the developed new product is natural with a clean label.

## Figures and Tables

**Table 1:** L*, a*, and b* color parameters of vegetable crispbreads based on beetroot (B) pomace and linseed (L).

Sample	L*	a*	b*
**100B**	32.82 ± 1.90 ^a^	21.16 ± 1.32 ^c^	11.54 ± 1.42 ^a^
**50B_50Lg**	39.41 ± 1.37 ^b^	16.66 ± 0.62 ^b^	23.15 ± 0.71 ^c^
**50B_50Ls**	37.93 ± 2.26 ^b^	18.01 ± 1.62 ^b^	15.90 ± 1.74 ^b^
**50B_25Lg_25Ls**	45.03 ± 2.76 ^c^	17.71 ± 1.46 ^b^	16.30 ± 2.26 ^b^
**100L**	53.30 ± 1.75 ^d^	7.97 ± 0.55 ^a^	24.72 ± 1.09 ^c^

Mean values ± standard deviations. Different superscript letters (^a–d^) within the same column indicate significant differences between the crispbreads (*p* < 0.05).

**Table 2:** Dry matter of vegetable crispbreads based on beetroot (B) pomace and linseed (L).

Sample	Dry Matter (%)
**100B**	93.43 ± 0.48 ^a^
**50B_50Lg**	97.53 ± 0.20 ^b^
**50B_50Ls**	97.04 ± 0.08 ^b^
**50B_25Lg_25Ls**	97.40 ± 0.06 ^b^
**100L**	97.10 ± 1.41 ^b^

Mean values ± standard deviations. Different superscript letters (^a,b^) within the same column indicate significant differences between the crispbreads (*p* < 0.05).

**Table 3:** Water activity of vegetable crispbreads based on beetroot (B) pomace and linseed (L).

Sample	Water Activity before Baking (−)	Water Activity after Baking (−)
**100B**	0.995 ± 0.000 ^a^	0.395 ± 0.001 ^d^
**50B_50Lg**	0.995 ± 0.000 ^a^	0.290 ± 0.009 ^a^
**50B_50Ls**	0.997 ± 0.002 ^a^	0.342 ± 0.003 ^c^
**50B_25Lg_25Ls**	0.994 ± 0.002 ^a^	0.310 ± 0.002 ^b^
**100L**	0.997 ± 0.003 ^a^	0.317 ± 0.005 ^b^

Mean values ± standard deviations. Different superscript letters (^a–d^) within the same column indicate significant differences between the crispbreads (*p* < 0.05).

**Table 4:** The water contact angle of vegetable crispbreads is based on beetroot (B) pomace and linseed (L).

Sample	0 s	5 s
**100B**	105.93 ± 4.5 ^c^	75.6 ± 4.21 ^a^
**50B_50Lg**	92.10 ± 5.13 ^b^	86.86 ± 7.50 ^b^
**50B_50Ls**	92.86 ± 3.65 ^b^	91.21 ± 3.54 ^b,c^
**50B_25Lg_25Ls**	101.22 ± 4.97 ^c^	98.05 ± 5.44 ^c^
**100L**	78.66 ± 6.83 ^a^	74.76 ± 7.17 ^a^

Mean values ± standard deviations. Different superscript letters (^a–c^) within the same column indicate significant differences between the crispbreads (*p* < 0.05).

**Table 5:** Texture of vegetable crispbreads based on beetroot (B) pomace and linseed (L).

Sample	Texture (N)
**100B**	7.80 ± 2.03 ^a^
**50B_50Lg**	13.83 ± 1.08 ^b^
**50B_50Ls**	8.08 ± 1.37 ^a^
**50B_25Lg_25Ls**	12.61 ± 2.06 ^b^
**100L**	18.86 ± 1.77 ^c^

Mean values ± standard deviations. Different superscript letters (^a–c^) within the same column indicate significant differences between the crispbreads (*p* < 0.05).

**Table 6:** Temperature and weight loss related to stages of TG/DTG curves of vegetable crispbreads based on beetroot (B) pomace and linseed (L).

Sample	First Stage		Second Stage		Third Stage		Fourth Stage		Fifth Stage	
	T (°)	Weight Loss (%)	T (°)	Weight Loss (%)	T (°)	Weight Loss (%)	T (°)	Weight Loss (%)	T (°)	Weight Loss (%)
**100B**	69.59	3.16	151.44 201.41 256.70	36.89	310.63	16.33	-	10.58	-	1.95
**50B_50Lg**	74.89	2.04	204.71 256.75	18.89	316.82	17.28	387.95	39.16	-	1.51
**50B_50Ls**	74.65	1.89	203.35 254.80	18.34	314.47	18.11	387.84	38.16	-	1.51
**50B_25Ls_25Lg**	74.12	1.68	206.66 257.99	14.93	316.54	18.14	390.77	44.67	-	1.41
**100L**	70.00	1.78	260.35	10.09	306.99	18.60	393.70	51.97	-	1.25

**Table 7:** Content of betalains in crispbreads and beetroot pomace of vegetable crispbreads based on beetroot (B) pomace and linseed (L).

Sample	Red Pigment Content (mg Betanin/100 g d.m.)	Yellow Pigment Content (mg Vulgaxanthin/100 g d.m.)
**100B**	51.44 ± 2.77 ^b^	171.13 ± 7.16 ^d^
**50B_50Lg**	14.59 ± 1.38 ^a^	51.31 ± 0.45 ^b^
**50B_50Ls**	16.62 ± 1.94 ^a^	50.02 ± 3.59 ^b^
**50B_25Lg_25Ls**	15.18 ± 0.07 ^a^	50.44 ± 1.17 ^b^
**100L**	3.87 ± 1.65 ^a^	12.29 ± 3.59 ^a^
**Fresh beetroot pomace**	241.14 ± 25.96 ^c^	121.63 ± 8.23 ^c^

Mean values ± standard deviations. Different superscript letters (^a–d^) within the same column indicate significant differences between the crispbreads (*p* < 0.05).

**Table 8:** Total phenolic content of vegetable crispbreads based on beetroot (B) pomace and linseed (L).

Sample	Total Phenolic Content (mg Chlorogenic Acid/100 g d.m.)
**100B**	820 ± 27 ^a^
**50B_50Lg**	730 ± 26 ^a^
**50B_50Ls**	830 ± 17 ^a^
**50B_25Lg_25Ls**	800 ± 51 ^a^
**100L**	948 ± 72 ^b^

Mean values ± standard deviations. Different superscript letters (^a,b^) within the same column indicate significant differences between the crispbreads (*p* < 0.05).

**Table 9:** Antioxidant activity of vegetable crispbreads based on beetroot (B) pomace and linseed (L).

Sample	ABTS (mg Trolox/g d.m.)	DPPH (mg Trolox/g d.m.)	Reducing Power (mg Trolox/g d.m.)
**100B**	2.58 ± 0.05 ^b^	2.41 ± 0.02 ^b^	7.90 ± 0.1 ^a^
**50B_50Lg**	1.46 ± 0.07 ^a^	2.48 ± 0.14 ^b^	7.80 ± 0.16 ^a^
**50B_50Ls**	1.82 ± 0.38 ^a^	2.31 ± 0.01 ^a,b^	8.94 ± 0.35 ^b^
**50B_25Lg_25Ls**	1.48 ± 0.07 ^a^	2.48 ± 0.02 ^b^	8.61 ± 0.55 ^a,b^
**100L**	1.88 ± 0.38 ^a^	2.20 ± 0.01 ^a^	10.00 ± 0.29 ^c^

Mean values ± standard deviations. Different superscript letters (^a–c^) within the same column indicate significant differences between the crispbreads (*p* < 0.05).

## Data Availability

The data presented in this study are available on request from the corresponding author.
